# Instructions for Flow Cytometric Detection of ASC Specks as a Readout of Inflammasome Activation in Human Blood

**DOI:** 10.3390/cells10112880

**Published:** 2021-10-26

**Authors:** Nico Wittmann, Ann-Kathrin Behrendt, Neha Mishra, Lukas Bossaller, Almut Meyer-Bahlburg

**Affiliations:** 1 Pediatric Rheumatology, Department Pediatric and Adolescent Medicine, University Medicine, University of Greifswald, 17489 Greifswald, Germany; nico.wittmann@med.uni-greifswald.de (N.W.); Ann-Kathrin.Behrendt@med.uni-greifswald.de (A.-K.B.); 2 Section of Rheumatology, Department of Medicine A, University Medicine, University of Greifswald, 17489 Greifswald, Germany; Neha.Mishra@med.uni-greifswald.de

**Keywords:** inflammasome, ASC (apoptosis-associated speck-like protein containing a caspase-recruitment domain), flow cytometry, human, PBMC, clinical study

## Abstract

Inflammasome activation is linked to the aggregation of the adaptor protein ASC into a multiprotein complex, known as the ASC speck. Redistribution of cytosolic ASC to this complex has been widely used as a readout for inflammasome activation and precedes the downstream proteolytic release of the proinflammatory cytokines, IL-1β and IL-18. Although inflammasomes are important for many diseases such as periodic fever syndromes, COVID-19, gout, sepsis, atherosclerosis and Alzheimer’s disease, only a little knowledge exists on the precise and cell type specific occurrence of inflammasome activation in patient samples ex vivo. In this report, we provide detailed information about the optimal conditions to reliably identify inflammasome activated monocytes by ASC speck formation using a modified flow cytometric method introduced by Sester et al. in 2015. Since no protocol for optimal sample processing exists, we tested human blood samples for various conditions including anticoagulant, time and temperature, the effect of one freeze–thaw cycle for PBMC storage, and the fast generation of a positive control. We believe that this flow cytometric protocol will help researchers to perform high quality translational research in multicenter studies, and therefore provide a basis for investigating the role of the inflammasome in the pathogenesis of various diseases.

## 1. Introduction

Inflammasomes are cytosolic multiprotein complexes that are assembled in response to various pathogen-associated molecular patterns (PAMPs) of bacterial or viral origin [1,2,3,4,5] as well as endogenous danger-associated molecular patterns (DAMPs) such as uric acid crystals or extracellular ATP [6,7,8,9]. A hallmark of the canonical inflammasome activation is the formation of large intracellular protein complexes composed of inflammasome sensors NAIP/NLRC4, NLRP3/6/7, AIM2/IFI16, and a central adaptor protein called ASC (apoptosis-associated speck-like protein containing a caspase activating and recruitment domain). The adapter protein ASC is rapidly recruited by the homotypic Pyrin–Pyrin domain interaction to the inflammasome sensors and assembles into large helical fibrils forming a supramolecular structure of 0.05–0.1 µm size, called the ASC speck [10,11,12,13,14]. Inflammatory caspases are then recruited via their CARD domain and undergo an autocatalytic activation which allows them to cleave pro-IL-1β and pro-IL-18 into the respective active pro-inflammatory cytokine forms [15].

Most of what we have learned in the last two decades about inflammasome biology results from cell culture and animal experiments. However, research to translate this knowledge into a clinical setting is still scarce. To date, the identification of inflammasome activated cells ex vivo from human samples has not been employed on a greater scale although inflammasomes are implicated in many clinically relevant diseases such as autoinflammatory periodic fever syndromes including cryopyrin-associated periodic syndromes (CAPS), familial mediterranean fever (FMF) or PAPA-syndrome [16,17,18], Alzheimer’s disease [19,20], and cancer [21,22,23], in addition to cardiovascular disorders [24], COVID-19 [25], diabetes [26]), gout [7,27], HIV-1 infection [28,29], sepsis [30], multiple sclerosis [31] and other neurological diseases [10,32,33]. 

Although often cited only a few groups have employed a flow cytometry based method introduced by Sester et al. [34] to identify inflammasome activated cells by ASC speck formation directly ex vivo [28,30,35,36,37]. The identification of inflammasome activated cells by flow cytometry allows the analysis of specific cell types and has several advantages compared to other methods such as microscopy (no reliable quantification of the number of activated cells) or immunoblotting (less sensitive, large number of cells required). Furthermore, human blood samples, for instance from children or other fluids such as bronchoalveolar lavage are limited in cell number and are thus often not sufficient to perform protein biochemistry.

Here we have therefore rigorously investigated the optimal conditions to reliably identify inflammasome activated cells by ASC speck formation ex vivo using flow cytometry. We provide detailed information on sample handling, factors contributing to falsified results as well as the optimal time corridor to perform the assay secondary to venipuncture and the generation of a fast positive control.

## 2. Materials and Methods

### 2.1. Reagents

The following reagents and chemicals were used: ultrapure LPS (Invitrogen, Waltham, MA, USA), ATP, DMSO, glucose, nigericin (Nig), trypan blue (all Sigma-Aldrich, St. Louis, MO, USA), Cytofix/Cytoperm™ Fixation/Permeabilization Kit (BD Bioscience, Franklin Lakes, NJ, USA), EDTA (Invitrogen), fetal calf serum (FCS), Ficoll, HEPES, PBS, Penicillin/Streptomycin, RPMI 1640 (all PAN-Biotech, Aidenbach, Germany), potassium chloride (Merck, Darmstadt, Germany) and β-mercaptoethanol (Thermo Fisher Scientific, Waltham, MA, USA). The following Antibodies (Abs) were used: rabbit polyclonal anti-ASC (AL177, Adipogen, San Diego, CA, USA), anti-rabbit-IgG-AlexaFluor488 (Invitrogen, Waltham, MA, USA), and lineage-specific antibodies CD3 PerCP, CD4 APC, CD14 APC-Cy7, CD16 PE-Cy7 (Biolegend, San Diego, CA, USA).

Reagents for immunofluorescence microscopy were: methanol (J.T. Baker, Phillipsburg, NJ, USA), bovine serum albumin (BSA) (Serva, Heidelberg, Germany), saponin, sodium pyruvate (Sigma-Aldrich, St. Louis, MO, USA), Image-iT™ FX Signal Enhancer and ProLong™ Diamond Antifade Mountant with DAPI (Invitrogen), and the following Abs were used: rabbit anti-ASC (AL177, Adipogen), mouse anti-beta-actin (Sigma-Aldrich), anti-rabbit-IgG-AlexaFluor488 (Invitrogen) and anti-mouse-IgG-AlexaFluor594 (Dianova, Hamburg, Germany).

### 2.2. Blood Sample Processing

All healthy donors were recruited at the Greifswald University of Medicine and included in the study after written informed consent. The study was conducted in accordance with the Declaration of Helsinki, and the protocol was approved by the local ethics committee (BB032/21). We used freshly donated whole blood from five different healthy donors for all experiments throughout the study. Peripheral blood mononuclear cells (PBMCs) were isolated using density gradient centrifugation. Briefly, after centrifugation for 10 min at 400 g, the plasma was removed and the remaining blood was diluted 1:1 with PBS, and carefully pipetted on the ficoll and centrifuged for 20 min at 460 g in a swinging-bucket rotor with a low brake applied. The PBMC interface was removed by pipetting and the cells washed with PBS by centrifugation for 10 min at 400 g and followed by a washing step at 320 g. The pellet was re-suspended and the cell count was calculated by using a trypan blue and a Neubauer counting chamber.

### 2.3. Freezing and Thawing Cells

After isolation, PBMCs were centrifuged and re-suspended in FCS + 10% DMSO. PBMCs were slowly frozen away at −80 °C, using a Mr. Frosty™ freezing container (ThermoFisher Scientific, Waltham, MA, USA) and transferred on the next day into the gas phase of a liquid nitrogen container. Cells were kept there for at least 4 weeks, before thawing them. For thawing, cells were warmed up fast and washed twice with PBS + 2 mM EDTA + 2% FCS. Cells were fixated with Cytofix™ (BD Bioscience) and stored for flow cytometry staining.

### 2.4. Cell Culture

The monocytic cell line THP1 was cultured in sterile RPMI 1640 supplemented with 10% FCS; 0.05 mM β-mercaptoethanol; 25 mM glucose; 10 mM HEPES; 1 mM sodium pyruvate; and 1:100 Penicillin/Streptomycin (10.000 U/mL/10 mg/mL) at 37 °C. THP1 and PBMC cells were stimulated either with 100 ng/mL LPS for 4 h, 10 µM Nig or 5 mmol/L ATP for 20 min, or with LPS for 4 h followed by Nig or ATP stimulation for 20 min in RPMI 1640 or PBS at 37 °C.

### 2.5. Flow Cytometry

For flow cytometry analysis, freshly isolated PBMCs or THP1 cells or frozen and thawed PBMCs as described were used. Cells with or without stimulation were fixed with Cytofix™ (BD Bioscience) on ice according to the manufacturer instructions. After washing with the permeabilization medium “Cytoperm™” (BD Bioscience), fixed samples were kept at 4 °C in a permeabilization medium overnight. For longer storage of fixed cells, we recommend a washing step and storage of samples in PBS. The next day cells were stained with rabbit polyclonal anti-ASC (1:1000) for 1 h on ice. After washing with 1 mL of permeabilization medium at 600 g for 8 min, supernatant was removed and cells were incubated on ice for 30 min with a permeabilization medium containing anti-rabbit-IgG-AlexaFluor488 (1:750) and lineage-specific antibodies CD3 PerCP, CD4 APC, CD14 APC-Cy7, CD16 PE-Cy7 (1:200). Cells were washed again with 1 mL permeabilization medium at 600 g for 8 min and flow cytometry was performed on a BD FACS Canto II (BD Biosciences). Samples were gated with the FlowJo software (FlowJo) to exclude debris (forward light scatter (FSC)-area versus side scatter-area (SSC)) and cell doublets were excluded using FSC-area versus FSC-height analysis. For THP1 cells, all single cells were considered for their ASC signal (ASC area vs. ASC width). Single PBMCs were further gated into CD3^−^ cells and then into CD14^++^CD16^−^ monocytes, which were analyzed for the ASC signal (ASC area vs. ASC width) (Figure S1).

### 2.6. Immunocytochemistry and Fluorescence Microscopy

To deposit cells into a clearly defined area of a “SuperFrost^®^ plus” glass slide (R. Langenbrinck, Emmendingen, Germany) the Shandon cytospin 4 (Thermo Scientific) was used. Cells were centrifuged and subjected to a slide while maintaining cellular integrity by medium acceleration of 1000 rpm for 5 min. Fixation was done with ice cold methanol at −20 °C for 15 min. The glass slides were stored at −80 °C until further usage. For staining, glass slides were warmed up fast and washed three times with an immunofluorescence buffer (PBS containing 0.2% BSA, 0.05% saponin, and 0.1% NaN_3_). To prevent flooding, cells were edged with a liquid-repellent slide marker (Plano GmbH), before incubating with Image-iT™ FX Signal Enhancer (Invitrogen) at room temperature (RT) for 30 min. After washing for 5 min on a tilt shaker with an immunofluorescence buffer, the incubation with rabbit anti-ASC (1:500) and mouse anti-beta-actin Ab (1:200) took place at 4 °C in a humid environment overnight. Slides were washed three times on a tilt shaker with an immunofluorescence buffer and incubated with anti-rabbit-IgG-AlexaFluor488 (1:750) and anti-mouse-IgG AlexaFluor594 (1:400) in the dark at RT for 1 h. The glass slides were washed again three times and coverslips were mounted on slides with the ProLong™ Diamond Antifade Mountant with DAPI (Invitrogen). Detection was performed on an Echo revolve hybrid microscope (Echo, version 3.1.1, San Diego, CA, USA). Overlay figures and a scale bar were generated with the Echo software (Echo).

### 2.7. Cytokine Determination

Isolated PBMCs from three healthy donors were stimulated in RPMI 1640 or PBS with either 100 ng/mL LPS for 4 h, 10 µM Nig for 20 min, 100 ng/mL LPS 4 h + 10 µM Nig 20 min, or left unstimulated. The supernatant was removed, frozen, and thawed for the analysis in a Legendplex (13-plex) Human Inflammation Panel 1 (Biolegend). The following cytokines were examined: IL-1β, IFN-α2, IFN-γ, TNF-α, MCP-1 (CCL2), IL-6, IL-8 (CXCL8), IL-10, IL-12p70, IL-17A, IL-18, IL-23, and IL-33. Samples were diluted in an assay buffer 1:2 and incubated with the beads at 4 °C overnight and for 1 h at RT on the next day. Further work steps were performed according to the manufacturer’s instructions. Samples were measured on a FACS Canto II and evaluated with the LEGENDplex™ Data Analysis Software (Biolegend).

### 2.8. Statistical Analysis

Data are presented as the mean + SEM from the number of assays indicated. Each data point is the mean value of measured triplicates. Statistics were calculated with Prism software (GraphPad, San Diego, CA, USA). A paired *t*-test was performed as appropriate. Multiple comparisons were analyzed by a repeated measure one-way or a two-way ANOVA, followed by Bonferroni multiple comparison test. A *p*-Value of * *p* < 0.05; ** *p* < 0.01 or *** *p* < 0.001 was considered significant.

## 3. Results

### 3.1. Flow Cytometric Detection of Spontaneous Intracellular ASC Specks Is Dependent on Anticoagulant, Storage Time, and Temperature

The development of a reliable method to visualize ASC speck formation by flow cytometry on a single cell level was published by Sester et al. in 2015 [34]. Under resting conditions, the inflammasome adaptor ASC is distributed uniformly all over the cell and rearranges under inflammasome activation into a large speck. We employed Sester’s method using a different polyclonal anti-ASC antibody (Ab) (AL177, Adipogen), since the original Ab (anti-ASC N-15, SantaCruz) is no longer commercially available. The anti-ASC Ab AL177 was used throughout the study at a concentration of 1:1000 following titrating experiments on LPS-primed and ATP or nigericin (Nig) stimulated PBMCs. As expected, we observed ASC speck forming CD14^++^CD16^−^ monocytes following inflammasome activation via a decrease in ASC fluorescent pulse width and a increase in ASC fluorescent pulse area [34] (Figure S1).

**Figure 1:**
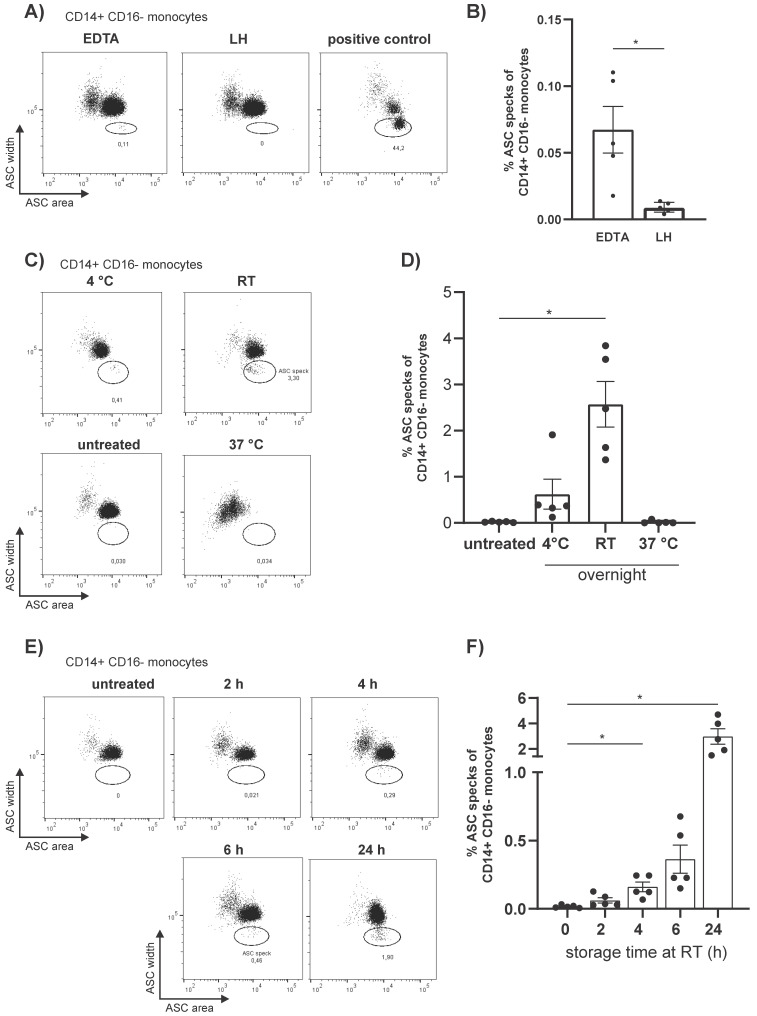
The choice of anticoagulant, storage temperature, and storage time of whole blood impact the unspecific formation of ASC speck^+^ cells in unstimulated monocytes. (**A**) Representative dot plots and (**B**) quantification of data of CD14^++^CD16^−^ monocytes for intracellular ASC pulse width vs. area isolated from EDTA- and LH blood in A) compared to LPS 4 h and Nig 20 min stimulated PBMCs. (**C**) Representative dot plots and (**D**) quantification of data of CD14^++^CD16^−^ monocytes isolated either directly after blood draw (untreated) or after storage of blood for 24 h at 4 °C, RT, or 37 °C. (**E**) Representative dot plots and (**F**) quantification of data of CD14^++^CD16^−^ monocytes isolated either directly after blood draw or after 2 h, 4 h, 6 h, or 24 h at RT. For statistics, paired two tailed t-test was used for (**B**) and repeated measure one-way ANOVA, followed by Bonferroni multiple comparison test for (**D**,**F**). *p*-Value * *p* < 0.05 was considered significant (*n* = 5 measured as triplicates and represented as the mean value from these, from three independent experiments, shown mean ± SEM).

Once the anti-ASC AL177 Ab was validated for flow cytometric detection of ASC specks we explored the optimal conditions for the processing of human blood samples to avoid unspecific ASC speck formation. We found that the type of anticoagulant in the blood collection tubes has an impact on the level of spontaneous ASC speck^+^ monocytes by flow cytometry (Figure 1A). Significantly higher numbers of unspecific ASC speck^+^ monocytes were detected in PBMCs collected from tubes with ethylenediaminetetraacetic acid (EDTA) compared with lithium heparin (LH) (Figure 1B). Therefore, due to the higher levels of unspecific ASC speck formation, the collection of blood in EDTA was rejected and LH was the anticoagulant of choice for further experiments.

Since clinical studies are often performed as multicenter studies, we wanted to test the optimal sample processing conditions for lithium heparin collected whole blood. To analyze the impact of the sample storage temperature we stored whole blood from healthy donors for up to 24 hours at 4 °C, room temperature (RT), and 37 °C. Subsequently PBMCs were isolated via density gradient centrifugation. Best results for PBMC isolation were obtained from blood stored at RT (Figure S2A). In contrast, storage at 4 °C resulted in severe cell clumping, whereas in blood stored at 37 °C large amounts of erythrocytes were found in the PBMC interface (Figure S2A). PBMCs from blood stored at 37 °C showed no spontaneous ASC specks and a generally weaker ASC staining of the CD14^++^CD16^−^ monocytes compared to the other conditions (Figure 1C). Storage at 4 °C for 24 h resulted in the spontaneous formation of ASC specks in CD14^++^CD16^−^ monocytes which were even more pronounced at RT compared to immediately isolated and fixed cells (Figure 1D).

Considering that we observed spontaneous ASC speck formation in unstimulated CD14^++^CD16^−^ monocytes from overnight stored LH-blood, but not in PBMCs isolated from freshly drawn blood, we determined the storage time until the formation of ASC specks occurs spontaneously. Since sample processing at 4 °C and 37 °C presented the aforementioned difficulties and sample transport under these temperatures is demanding, we stored whole blood from healthy donors at RT for 0 h, 2 h, 4 h, 6 h, and 24 h. A significant increase in ASC speck^+^ CD14^++^CD16^−^ monocytes occurred already after 4 h storage at RT compared to directly processed samples (Figure 1F) and the number of ASC speck^+^ monocytes further accumulated over time. Hence, we recommend that PBMCs should be isolated within 2 hours after blood collection to avoid unspecific ASC speck formation.

### 3.2. Storage of PBMCs in Liquid Nitrogen Does Not Impact Spontaneous ASC Speck Formation

**Figure 2:**
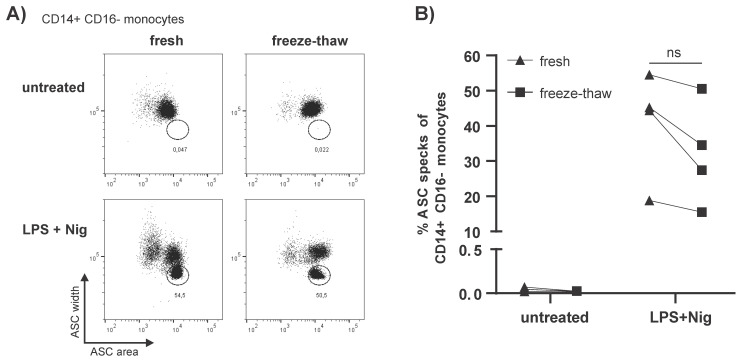
**Isolated PBMCs can be frozen for long-term storage without spontaneous ASC speck formation.** (**A**,**B**) ASC speck formation in CD14^++^CD16^−^ monocytes either freshly isolated or after one freeze–thaw cycle with or without stimulation with LPS 4 h and Nig 20 min. (**A**) Representative dot plots and (**B**) quantification of data tested with two-way ANOVA followed by Bonferroni multiple comparison test (*n* = 4, individual frozen away and thawed together). ns: no significance.

Next, we wanted to know if a single freeze–thaw cycle and storage in liquid nitrogen following the isolation of PBMCs would influence the occurrence of spontaneous ASC specks in monocytes. Therefore, unstimulated and stimulated PBMCs were frozen and stored for at least four weeks in liquid nitrogen. The PBMCs were subsequently thawed and spontaneous ASC speck formation analyzed by flow cytometry. No increase of unspecific ASC speck^+^ monocytes was observed after one freeze–thaw cycle as previously described [28]. However, in LPS primed and Nig stimulated PBMCs a trend towards fewer ASC speck^+^ cells after one freeze–thaw cycle was observed (Figure 2A,B).

### 3.3. Fast Generation of a Reliable ASC Speck Positive Control

Usually, a positive control for ASC speck formation is generated by priming PBMCs with LPS for several hours followed by a second stimulus with ATP or Nig. As this is a rather time-consuming method and thus may complicate routine procedures within a clinical trial, we wanted to establish a faster way to create a reliable positive control.

**Figure 3:**
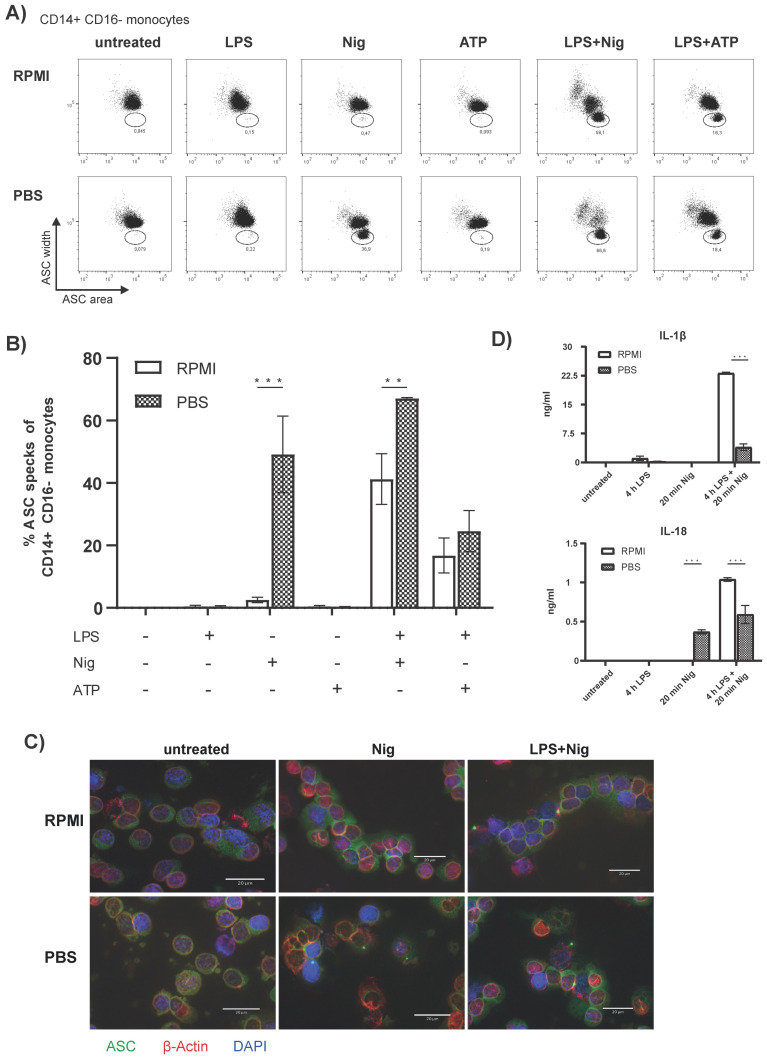
**Incubation of PBMCs with Nig in PBS leads to high numbers of ASC speck^+^ monocytes.** (**A**,**B**) ASC speck formation in CD14^++^CD16^−^ monocytes after stimulation as indicated or untreated (**A**) Representative dot plots and (**B**) Quantification of data. (**C**) Fluorescence microscopic images of ASC speck formation in untreated or stimulated PBMCs. ASC (green), β-actin + (red) and DAPI (nuclei, blue) (scale bar 20 µm). (**D**) Cytokine levels in the supernatant of untreated or stimulated PBMCs as indicated. Repeated measure one-way ANOVA for (**B**) and a two-way ANOVA for (**C**), followed by a Bonferroni multiple comparison test was performed. A *p*-Value ** *p* < 0.01; *** *p* < 0.001 was considered significant (*n* = 3 measured as triplicates and represented as the mean value from these, from four independent experiments for (**B**) and *n* = 3 measured in duplicates for (**D**), shown mean ± SEM).

A recent study demonstrates by western blot analysis that a priming step is not necessarily required for NLRP3 inflammasome activation in human monocytes in vitro [38]. We, therefore, investigated if we can recapitulate this via a flow cytometric analysis of ASC speck formation. We stimulated freshly isolated PBMCs with Nig or ATP with or without prior priming with LPS. In addition, we compared the standard way of cell culture in RPMI versus incubation of cells in PBS, since we were interested if residual PBS from isolating PBMCs intermixed with RPMI in culture may impact the further process of ASC speck formation. Surprisingly, incubation with Nig alone, but not ATP, in PBS resulted in a significant increase of ASC speck^+^ monocytes in PBMCs (Figure 3A,B). The number of ASC speck^+^ monocytes was comparable to the conventionally used method of LPS priming and inflammasome activation with Nig. Moreover, when we incubated LPS and Nig treated PBMCs in PBS compared to RPMI as a culture medium (Figure 3A,B) we observed even higher numbers of ASC speck^+^ monocytes.

To further confirm the formation of ASC specks by incubating with Nig alone without prior LPS priming in PBS, we used widefield fluorescence microscopy. Under resting conditions, endogenous ASC was homogeneously distributed over the cell. Upon inflammasome activation with Nig alone or a combination of LPS and Nig the ASC rearranges into clearly visible ASC specks (Figure 3C). Using widefield fluorescence microscopy the ASC specks from cells incubated with Nig and PBS appeared similar to the ASC specks in LPS primed and Nig stimulated cells.

Activation of the NLRP3 inflammasome activates caspase 1 which subsequently leads to the secretion of active IL-1β and IL-18. We, therefore, wanted to test if incubation of PBMCs with Nig alone not only results in ASC speck formation but also to inflammasome induced cytokine secretion. A significant increase in IL-18 was observed in the supernatant of PBMCs stimulated in PBS with Nig with or without prior LPS priming (Figure 3D). In contrast, IL-1β was detectable in LPS primed and Nig stimulated cells but not after incubation with Nig alone. Similarly, IL-6 and IL-8 were increased in LPS primed and Nig stimulated PBMCs and generally higher amounts were secreted in RPMI compared to PBS (paired *t*-test, Supplementary Figure S3).

**Figure 4:**
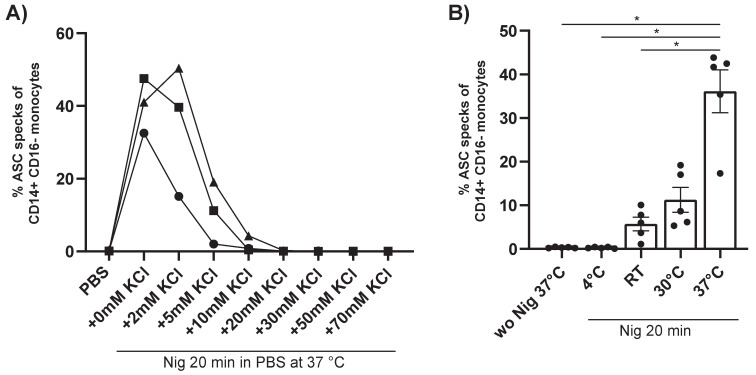
**ASC speck formation when incubating with Nig in PBS is dependent on K^+^ concentration and temperature.** (**A**) Percentage of ASC speck^+^ monocytes of CD14^++^CD16^−^ monocytes from PBMCs left untreated or incubated with Nig in PBS. Before adding the Nig, PBS was supplemented with different concentrations of KCl. (**B**) Percentage of ASC speck^+^ monocytes of CD14^++^CD16^−^ monocytes from PBMCs left untreated or incubated with Nig in PBS at 4 °C, RT, 30 °C, or 37 °C for 20 min. Repeated measure one way-ANOVA, followed by a Bonferroni multiple comparison test was performed. A *p*-Value * *p* < 0.05, was considered significant (*n* = 3 for (**A**) and *n* = 5 for (**B**) measured as triplicates and represented as the mean value from these, from three different experiments, shown mean ± SEM).

The unexpected finding that stimulation of PBMCs with Nig alone in PBS, but not in RPMI leads to spontaneous ASC speck formation could be due to a different electrolyte concentration of the two media. In particular, potassium has been shown to play an important role in ASC speck formation. Therefore, we tested the effect of the gradual addition of KCl. The results show that ASC speck formation following stimulation with Nig in PBS, without prior LPS priming is gradually inhibited by potassium (Figure 4A). Addition of KCl into PBS before stimulating the cells with Nig at a concentration of 30 mM KCl completely blocked the formation of ASC speck^+^ monocytes. Subsequently, we also tested the impact of temperature. Higher temperature led to increased ASC speck formation (Figure 4B). We conclude that the strong ASC speck formation following stimulation with Nig in PBS, without prior LPS priming, is a potassium and temperature dependent process.

### 3.4. The Monocytic THP1 Cell Line Represents a Valid ASC Speck Positive Control

**Figure 5:**
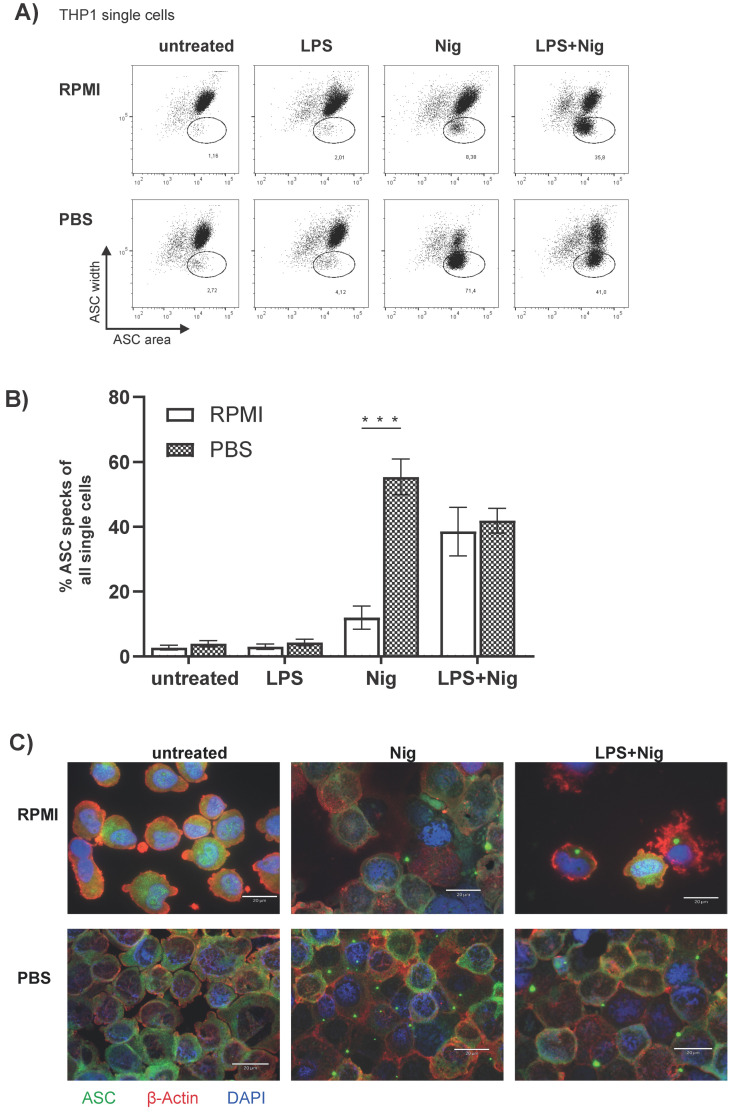
**Incubation with Nig without prior priming results in the formation of ASC speck^+^ cells in THP1 cells.** (**A**) Representative dot plots and (**B**) quantification of data of THP1 cells for intracellular ASC. THP1 cells were stimulated as indicated in (**A**). (**C**) Fluorescence microscopic images of ASC specks in THP1 cells stained as described in 3C) either left untreated or stimulated as indicated (scale bar 20 µm). A two-way ANOVA, followed by a Bonferroni multiple comparison test was performed. A *p*-Value *** *p* < 0.001 was considered significant (*n* = 4 measured as triplicates and represented as the mean value from these, from four independent experiments, shown mean ± SEM).

Monocytes represent a relatively small population (2–14% of the leukocytes) and are thus not easy to isolate in sufficient amounts for mechanistical and functional experiments. Therefore, the monocytic THP1 cell line is often used as an alternative in vitro model for CD14^++^ CD16^−^ monocytes. We therefore analyzed if Nig alone in PBS results in ASC speck formation in THP1 cells as well. First, stimulated and unstimulated THP1 cells were investigated by flow cytometry for ASC speck formation. Compared to monocytes directly analyzed ex vivo, cultured THP-1 cells exhibit a low basal rate of spontaneous ASC speck formation. Similar to freshly isolated human PBMCs, THP1 cells incubated with Nig in PBS compared to stimulated cells in RPMI show a significant increase in ASC speck^+^ cells (Figure 5A,B). In PBMCs we observed a marked difference between RPMI versus PBS incubated and LPS and Nig stimulated ASC speck^+^ monocytes (Figure 3B) with more ASC speck^+^ monocytes in the PBS condition; however, in THP-1 cells we did not observe this difference (Figure 5B). ASC specks can be detected in THP1 cells stimulated with Nig in RPMI as well as in PBS (Figure 5C). The ASC specks from cells incubated with Nig and PBS appeared similar to the conventional ASC specks in LPS primed and Nig stimulated cells.

## 4. Discussion

Investigating inflammasome activation in clinical studies is a challenging endeavor. Many studies trying to close the gap between basic inflammasome research and clinically relevant questions concerning disease activity are limited by the employed methods. They either do not directly prove inflammasome activation, for instance if measuring mRNA expression of inflammasome components via qPCR, or they do not account for the fact that IL-1β or IL-18 can be released by other processes independent of inflammasome activation [39,40,41]. Lastly, some studies use ex vivo cell cultures of PBMCs to approximate inflammasome activation in the patients.

Therefore, to determine activation of inflammasomes directly from patient samples, the detection of ASC speck^+^ cells by flow cytometry is particularly suitable, since even small blood samples of approximately 2 mL are sufficient. Especially when compared to immunoblotting smaller numbers of cells are needed to determine inflammasome activation via ASC speck formation. Furthermore, flow cytometry is cell type specific. Beyond this, imaging flow cytometry can also be used to detect ASC specks in PBMCs [42]. This method combines conventional flow cytometry with microscopic images of each cell. Despite this useful feature, the method is not widely used because it is technically demanding, time-consuming, and requires an experienced investigator. However, imaging flow cytometry, as well as flow cytometry, has limitations for instance when analyzing tissue samples from inflammatory sites. Attempts to disrupt the tissue for later flow cytometry may destroy the fragile pyroptotic ASC speck^+^ cells.

In comparison to Sester’s paper we use a different antibody and focus on human PBMCs, especially on CD14^+^CD16^−^ monocytes, in the context on clinical applicability. In contrast, Sester et al. used PBMCs, bone marrow derived macrophages and HEK293 cells. We showed that pre-analytical handling can have a decisive influence on the results. To obtain reliable and reproducible high-quality data in multicenter clinical research studies optimal standard operating procedures are required. In the present report, we, therefore, identified important factors for the handling of human whole blood for detection of ASC specks by flow cytometry, including anticoagulant, temperature, and duration of storage. In addition, the fast generation of a positive control for ASC speck formation is described.

Many studies have shown that the choice of anticoagulant influences the result of the whole blood testing. This depends in particular on the cell population examined. For myeloid-derived suppressor cells (MDSC) it has been shown that K_2_EDTA leads to more consistent results compared to sodium–heparin [43]. For the performance of a monocyte monolayer assay the highest reliability was obtained using anticoagulant citrate dextrose (ACD) [44]. In a study from the Netherlands using the EuroFlow protocol for high dimension flow cytometry, expression of cell surface markers and population distribution were more stable in Na-Heparin blood than in EDTA blood, but storage of Na-Heparin samples was associated with faster decrease in leukocyte counts over time [45].

The use of K_2_EDTA blood collection tubes in our study resulted in a significant increase in ASC speck^+^ monocytes compared to LH. This might be due to the fact, that in the current study, we compared K_2_EDTA with lithium heparin (LH) blood collection tubes for blood sampling. EDTA acts as a chelator to bind free cations, such as Ca^2+^ ions from the blood, thus removing them from the coagulation cascade and prevents blood clotting. This can impact extracellular electrolyte concentrations. In contrast, LH binds the antithrombin in the blood and thereby enhances the inhibitory effect on the clotting factors such as thrombin or factor X [46]. We, therefore, recommend LH for blood samples when determining ASC speck formation.

The storage time is an even more important pre-analytical variable. The time of blood collection often cannot be planned well, especially when dealing with acutely ill patients. On the other hand, in many multicenter studies, the blood sample has to be sent to another location. A recent publication recommends storing whole blood rather at RT than cooling it at 4 °C for a longer period of time to minimize the negative effect on PBMCs [47]. We were able to confirm this observation, as PBMCs of sufficiently good quality could only be isolated from blood stored at RT (Figure S2A). However, storage of blood for 24 h at 37 °C resulted in marked contamination of the PBMC interface with a large number of erythrocytes which impeded subsequent immunostaining. We cannot exclude, that with a different isolation protocol, the purity of PBMCs could eventually be improved. However, for the current study, we decided to store blood samples at RT. This also seems more reasonable in the context of multicenter studies. In contrast, when blood samples were stored at 4 °C, we observed cell clumping in the PBMC interface as previously described by Jerram et al. [47] which might be caused by spontaneous platelet activation at 4 °C [48,49].

While blood storage at RT for 24 hours was sufficient for PBMC isolation, it resulted in a significant increase in unspecific ASC speck^+^ monocytes compared with immediately isolated PBMCs (Figure 1D). Spontaneous ASC speck formation could be already observed after 4 h of storage of whole blood at RT (Figure 1E,F).

Blood samples should ideally be processed by density gradient separation immediately after blood collection to isolate PBMCs or at least within 2 h to avoid spontaneous ASC speck formation. After isolating PBMCs they should be fixed immediately in a fixation buffer and stored at 4 °C until further processing for flow cytometry.

However, this is not always possible due to the location of clinical trial centers relative to research laboratories. Therefore, freezing and storage of PBMCs in liquid nitrogen could be an alternative. PBMCs could then be sent to the site of analysis in a frozen state at a later time. However, this requires that freezing does not impact ASC speck formation. We, therefore, tested the effect of freezing on ASC speck formation. After one freeze–thaw cycle, unstimulated cells showed no spontaneous ASC speck^+^ monocytes in unstimulated PBMCs. However, the number of ASC speck^+^ cells appeared to decrease with freezing (Figure 2A,B). Although not statistically significant, a trend towards fewer ASC speck^+^ cells was observed after the freeze–thaw cycle in LPS primed and Nig stimulated PBMCs. This is not unexpected since cells with an active inflammasome go into pyroptosis, which is a form of inflammatory programed cell death activated by human inflammatory caspases [50]. A freeze–thaw cycle is an additional stress signal and thus results in cell death. These cells would then no longer be detectable in the subsequent flow cytometric analysis and the number of ASC speck^+^ monocytes would be lower after thawing. This has to be kept in mind if the study design requires freezing of PBMCs to be transported between participating centers.

It is known that inflammasome activation is a highly regulated process and is only formed after a first priming step, as by stimulation with LPS, followed by a second activation signal. The priming step usually takes several hours. During this time, gene transcription for pro-inflammatory cytokines is upregulated via the NF-κB pathway [51,52]. After the priming step, the inflammasome can be activated by ATP or Nig [53,54]. However, there are several reports that have described inflammasome activation without a priming signal [55,56].

Remarkably, we observed a strong and highly significant increase in ASC speck^+^ monocytes after incubation of PBMCs with Nig alone for only 20 min without prior priming with LPS. (Figure 3A,B). This finding is consistent with a recent publication showing that activation with Nig alone is sufficient to activate the NLRP3 inflammasome, form ASC specks and cleave caspase 1 [38]. Interestingly, ASC speck formation by Nig alone could only be observed when cells were incubated in PBS instead of RPMI, and ATP stimulation alone did not result in a similar effect.

ATP acts through the P2X_7_ receptor, which promotes Ca^2+^ and Na^+^ influx and coordinates K^+^ efflux. In particular, K^+^ efflux is critical for inflammasome activation. On the other hand, Nig functions directly as an ionophore and causes the efflux of K^+^ ions [54,57,58,59,60,61]. We therefore assume that this effect is caused by differences in extracellular electrolyte concentrations, most importantly potassium and calcium. Consistent with this idea the increased number of ASC speck^+^ monocytes after stimulation with Nig in PBS is dependent on the extracellular potassium concentration. The addition of 30 mM KCl leads to a complete block of ASC speck formation (Figure 4A). For practical reasons we further confirmed that small quantities of PBS, which may still be present after density gradient centrifugation, do not affect stimulation with Nig (Supplementary Figure S2B).

ASC formation in monocytes appears to be functional even when PBMCs are incubated with Nig for 20 min in PBS, since IL-18 could be detected in the supernatant of stimulated PBMCs. In contrast, IL-1β levels were below detection level (Figure 3D) and therefore in a striking discrepancy to the vast amount of speck positive monocytes under this condition. We presume, that these findings are consistent with a recent publication demonstrating that pro-IL18 is permanently expressed in PBMCs and can be immediately processed into its biological active counterpart upon appropriate inflammasome formation [62]. However, pro-IL-1β has first to be synthesized and without LPS priming the NF-κB pathway gene transcription is not activated and therefore pro-IL-1β is not produced. Although we currently do not know the mechanism, why Nig in PBS induces a stronger ASC speck formation in monocytes compared to RPMI as a medium, we conclude, that incubation of PBMCs with Nig in PBS can be used as a fast and reliable positive control for ASC formation by flow cytometry.

The THP1 cell line, in addition to human and murine primary cells, is frequently used as a model cell line to study inflammatory issues. Both, the detection of ASC speck formation by flow cytometry and induction of ASC by stimulation with Nig alone without prior priming with LPS also works in THP1 cells (Figure 5A,B). The THP1 cell line, therefore, represents a reasonable in vitro model for NLRP3 inflammasome activation for mechanistical analyses.

## 5. Conclusions

The results of our study have shown the advantages of collecting blood samples in LH, as compared to EDTA, regarding unspecific ASC speck formation. It needs to be kept in mind that when blood samples are collected in EDTA, ASC speck^+^ cells are slightly increased. The LH blood should be stored at RT and isolation of PBMCs should start within 2 h after blood collection. Preferentially, PBMCs should be isolated directly following venipuncture and fixed immediately. If this is not possible, PBMCs can be frozen until further usage, without the formation of ASC specks due to nonspecific activation. However, freezing will cause some loss of ASC speck^+^ cells. For the fast generation of an ASC speck positive control, we recommend incubating PBMCs with Nig alone in PBS.

## Data Availability

Not applicable.

## Supplementary Materials

The following are available online at https://www.mdpi.com/article/10.3390/cells10112880/s1, Figure S1: Gating strategy, Figure S2: (A) Proper PBMC isolation is only possible in blood stored at RT; (B) Low levels of PBS in the RPMI medium do not cause ASC speck formation, when stimulated with Nig, Figure S3: Comparison of cytokines in the supernatant of LPS primed and Nig stimulated PBMCs in RPMI versus PBS.
