# Supplementary material for: Instructions for Flow Cytometric Detection of ASC Specks as a Readout of Inflammasome Activation in Human Blood
Source: Cells. 2021 Oct 26;10(11):2880. doi: 10.3390/cells10112880 (PMC8616555; doi:10.3390/cells10112880)
Supplement: Supplementary file 1 [file cells-10-02880-s001.zip › Supplementary figure legend.pdf]

S1. Gating strategy. Representative dot plots of PBMCs incubated with Nig. Backgating of ASC speck+ and ASC speck- cells.

S2. A) Proper PBMC isolation is only possible in blood stored at RT. Picture taken after density gradient centrifugation was performed with LH blood, which was stored for 24 h at 4 °C, RT or 37 °C. **B) Low levels of PBS in the RPMI medium do not cause ASC speck formation, when stimulated with Nig.** Isolated PBMCs were stimulated with Nig in RPMI and PBS alone or in a define mixture of both. Repeated measure one way-ANOVA, followed by a Bonferroni multiple comparison test was performed. A p-Value $*p<0.05$ was considered significant ($n = 3$ measured in single determination, from two different experiments, shown mean ± SEM).

S3. Comparison of cytokines in the supernatant of LPS primed and Nig stimulated PBMCs in RPMI versus PBS. Cytokines where detected in the supernatant of PBMCs stimulated with LPS 4 h and Nig 20 min in RPMI or PBS. The concentration of IL-8 in RPMI was above the highest standard and the value was equated to the highest standard value. Paired two tailed t-test was used for statistic comparison. A p-Value $*p<0.05$, $**p<0.01$ was considered significant ($n = 3$ measured in duplicates and represented as the mean value from these, shown mean ± SEM).
