# Supplementary figures and images for: Instructions for Flow Cytometric Detection of ASC Specks as a Readout of Inflammasome Activation in Human Blood
Source: Cells. 2021 Oct 26;10(11):2880. doi: 10.3390/cells10112880 (PMC8616555; doi:10.3390/cells10112880)

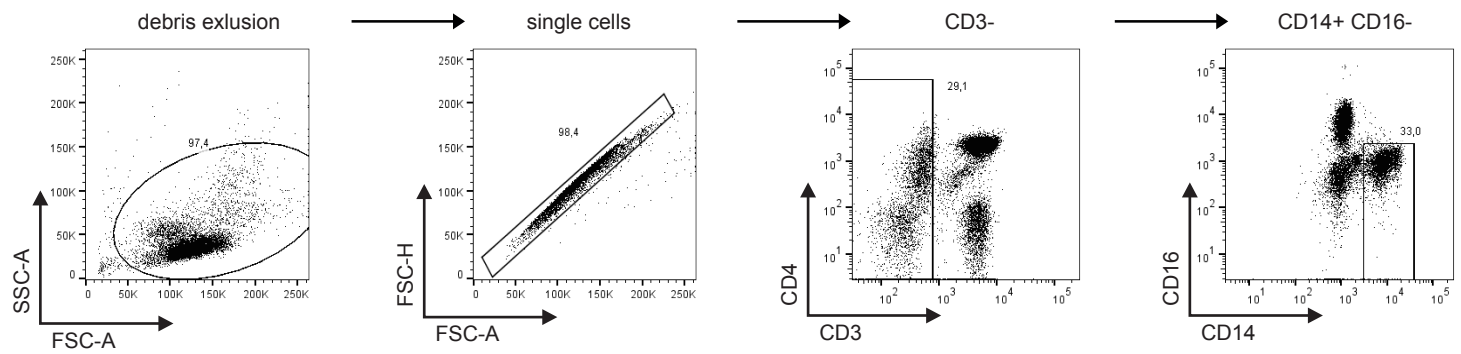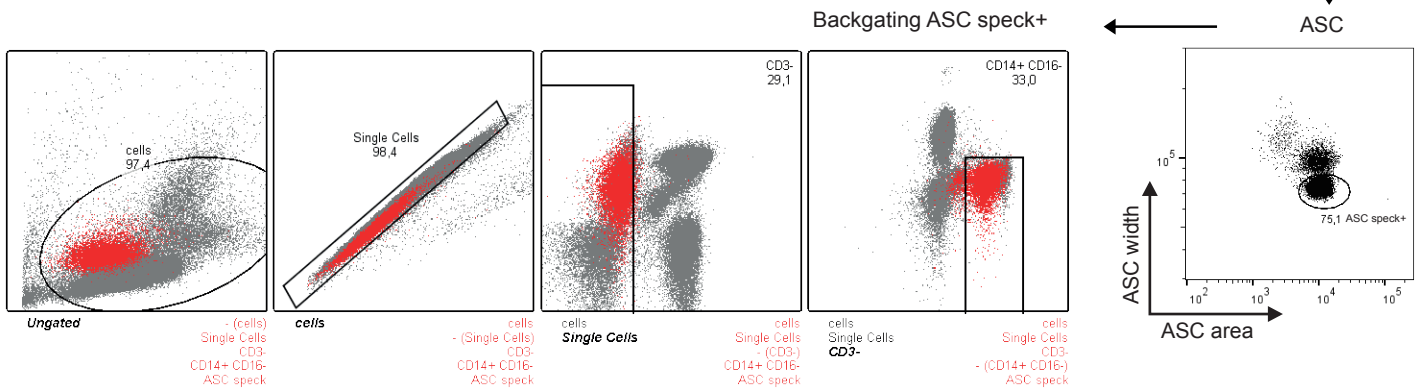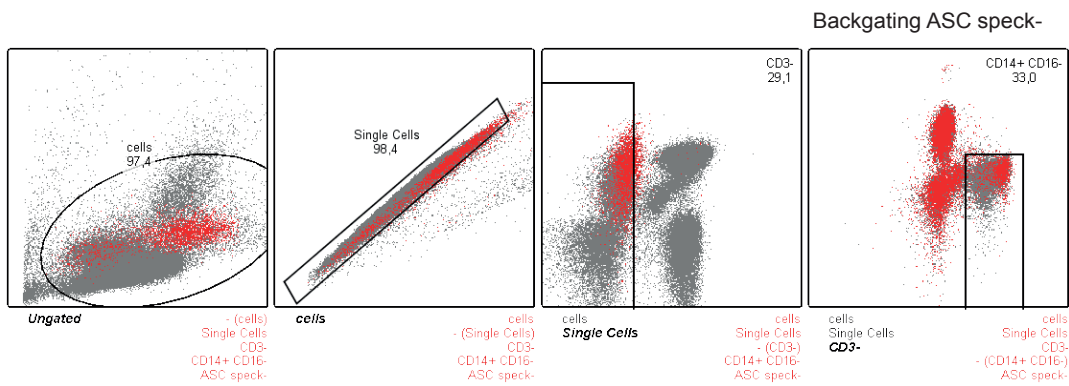

Supplement: Supplementary file 1 [file cells-10-02880-s001.zip › Figure S1.pdf]

A)

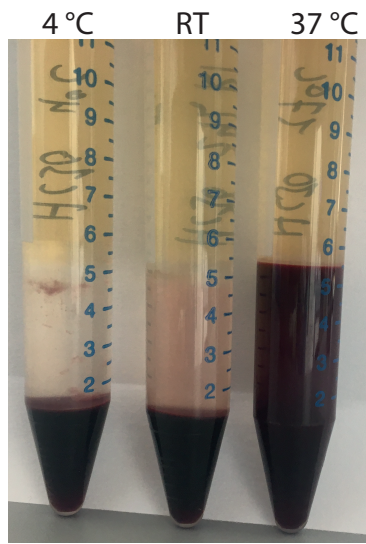

B)

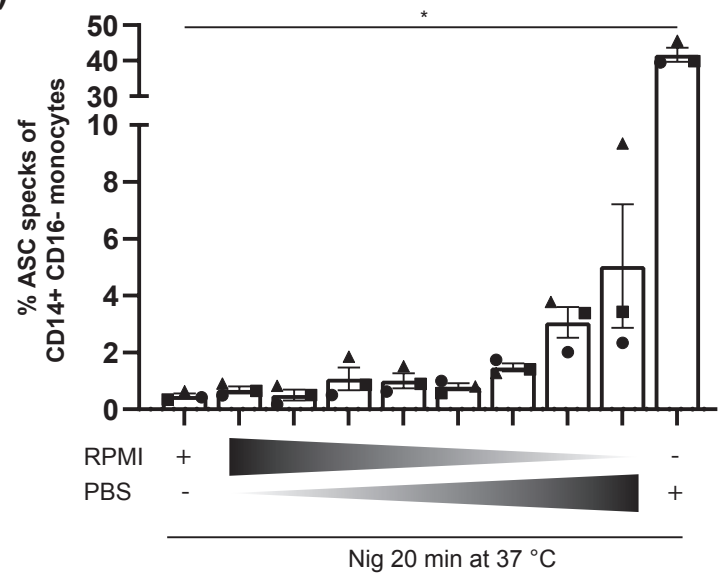

Supplement: Supplementary file 1 [file cells-10-02880-s001.zip › Figure S2.pdf]

supernatant of LPS primed 4 h and with Nig 20 min stimulated PBMCs

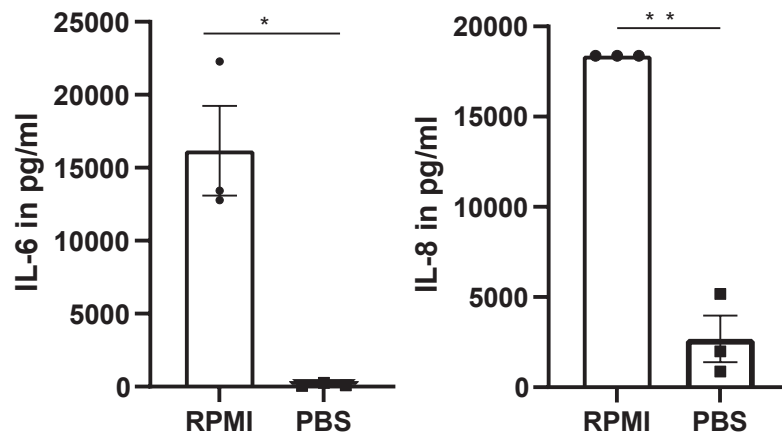

Supplement: Supplementary file 1 [file cells-10-02880-s001.zip › Figure S3.pdf]
